# Neurovascular, Structural, and Molecular Mechanisms of Cognitive Impairment in Type 2 Diabetes Mellitus: A Systematic Review

**DOI:** 10.7759/cureus.118011

**Published:** 2026-10-16

**Authors:** Meghna Dipakkumar Chaudhary, Bhanpratap Ahirwar, Keisha Shah, Sharanya Sreekanthaswamy, Naiya Bhavsar, Niraj Lodha, Karthika Padmavathy

**Affiliations:** 1 Dental Surgery, Karnavati University School of Dentistry, Gandhinagar, IND; 2 General Medicine, Virendra Kumar Saklecha Government Medical College, Neemuch, IND; 3 Internal Medicine, GMERS Medical College, Vadodara, IND; 4 Medicine and Surgery, Kempegowda Institute of Medical Sciences, Bangalore, IND; 5 Respiratory Medicine, Dr. N.D. Desai Faculty of Medical Science and Research, Dharmsinh Desai University, Nadiad, IND; 6 Internal Medicine, Lodha Hospital and Research Centre, Pali, IND; 7 Pathology, Sri Lalithambigai Medical College and Hospital, Dr. M.G.R. Educational and Research Institute, Chennai, IND

**Keywords:** cognitive impairment, diabetes mellitus, molecular mechanisms, neurovascular dysfunction, type 2 diabetes mellitus

## Abstract

Type 2 diabetes mellitus (T2DM) is associated with cognitive impairment and neurodegenerative changes in addition to its established metabolic and vascular complications. Evidence indicates that retinal and cerebral microvascular abnormalities, structural brain alterations, impaired neurovascular coupling, and metabolic and molecular disturbances may contribute to cognitive vulnerability in individuals with T2DM. This systematic review synthesized evidence on the neurovascular, structural, cognitive, metabolic, molecular, and genetic factors associated with cognitive impairment in T2DM. The review was conducted and reported in accordance with the Preferred Reporting Items for Systematic Reviews and Meta-Analyses (PRISMA) 2020 framework. A structured search of PubMed/MEDLINE, Scopus, Web of Science, and Google Scholar was performed using predefined eligibility criteria. Study selection and data extraction were undertaken independently by two reviewers, with disagreements resolved through discussion and consensus. Ten studies comprising cross-sectional, observational, neuroimaging, longitudinal, genetic, and Mendelian-randomization designs were included. Risk of bias was assessed using study-design-appropriate appraisal tools, and findings were synthesized narratively because of heterogeneity in study designs, populations, assessment methods, and outcome measures. The included studies reported retinal neurovascular abnormalities before clinically detectable diabetic retinopathy, gray- and white-matter volume alterations in individuals with T2DM and mild cognitive impairment, and longitudinal deterioration in neurovascular coupling associated with memory performance. Higher glycated hemoglobin levels and other vascular and metabolic factors were associated with poorer cognitive outcomes, while lower soluble low-density lipoprotein receptor-related protein 1 levels were associated with mild cognitive impairment in one biomarker study. Cognitive impairment was frequently reported among the studied T2DM populations; however, Mendelian-randomization evidence did not establish a direct causal association between T2DM and impaired cognition. Overall, the evidence suggests that cognitive impairment in T2DM is associated with interconnected microvascular, structural, metabolic, molecular, and genetic factors, although causal inference remains limited by the predominance of cross-sectional studies and methodological heterogeneity. Larger longitudinal and multimodal studies are needed to clarify temporal relationships, validate potential early biomarkers, and determine their clinical relevance.

## Introduction and background

Diabetes mellitus type 2 (T2DM) refers to an irreversible chronic metabolic disorder that leads to insulin resistance and uncontrolled hyperglycemia and has a vastly deleterious community health consequence on millions of people worldwide [1]. T2DM has been among the most pressing non-communicable conditions of the present-day world due to the ageing process, the establishment of a sedentary lifestyle, and the rise in the prevalence of obesity [2]. In addition to its vascular and metabolic complications, known long ago, T2DM has also turned out to be a significant risk factor for cognitive impairment and dementia [3]. T2DM is associated with mild cognitive impairment (MCI) and severe neurodegenerative disorders, such as Alzheimer's disease, as a cognitive impairment [4]. MCI is reportedly a transition between normal ageing of the mind and dementia, and a big proportion of the individuals with MCI subsequently develop dementia [5]. It is stated that T2DM patients tend to develop MCI more often than non-diabetic individuals, and that is why it is necessary to diagnose and treat the disease at an early stage [6].

The pathophysiology of the interaction between T2DM and cognitive decline is multifactorial, involving vascular, metabolic, and neurodegenerative mechanisms [7]. The long-term effects of hyperglycemia on the endothelium, oxidative stress, and inflammation are some of the factors that lead to the deterioration of cerebral blood flow and neuronal integrity [8]. The alterations may worsen neurovascular coupling and cause local damage to the areas of the brain involved in memory and executive functions [9]. The neuroimaging experiments have indicated that T2DM patients tend to experience structural changes in the brain that, besides the degeneration of the white matter, also incorporate the disappearance of the grey matter [10]. This is greater in regions such as the hippocampus and the prefrontal cortex, which play an important role in the thinking process [11]. Longitudinal studies have also shown that neurovascular damage and reduced cerebral perfusion may precede structural damage, which demonstrates that vascular damage has an insidious effect on cognitive impairment [12].

New developments also suggest retinal and microvascular alterations as the initial symptoms of neural impairment in diabetes [13]. The retina is functionally and structurally comparable to the brain, and retinal neurodegeneration may indicate the existence of larger neurovascular pathology [14]. The retinal lesions have been established to be capable of preceding the clinical retinopathy, and this has been in favour of their potential application as early biomarkers of impaired cognitive ability [15]. Other causes of cognitive decline in T2DM include metabolic and molecular factors. Low glycemic control, as commonly measured by glycated haemoglobin (HbA1c), has been identified again and again to be linked to cognitive impairment [1]. Insulin resistance and dysfunctional insulin signalling in the brain may cause neuronal metabolism and synaptic plasticity dysfunction, resulting in cognitive deficits [8]. In addition, the occurrence of advanced glycation end-products and high amounts of oxidative stress can lead to an increase in the pace of neurodegeneration [3].

A second issue is that of the role of amyloid metabolism and associated biomarkers. They lead to the development of Alzheimer's disease, and such proteins as low-density lipoprotein receptor-related protein 1 (LRP1) facilitate the clearance of the amyloid-2 protein [7]. Any change in such pathways can also relate T2DM to Alzheimer-like pathology, which also highlights the similarity between metabolic and neurodegenerative diseases [5]. Alternatively, it could be that the inclination to develop cognitive impairment is genetic in nature, which is also under investigation at the moment [6]. This is because the issue of T2DM is becoming increasingly burdensome, and the fact that the issue is associated with cognitive impairment necessitates a comprehensive description of the processes and the earliest indicators of the impairments [7]. Timely intervention and better clinical outcomes can be achieved through early detection of neurovascular and structural changes [8]. This systematic review aims to organise the body of knowledge on neurovascular, structural, and molecular mechanisms of cognitive impairment in T2DM. Previous reviews have established an association between T2DM and cognitive impairment; however, the available evidence remains fragmented across vascular, structural, metabolic, molecular, and genetic domains. In particular, retinal and cerebral microvascular abnormalities, neuroimaging findings, molecular biomarkers, and genetic factors have not been sufficiently integrated within a unified mechanistic framework. The predominance of cross-sectional studies further limits understanding of temporal relationships, disease progression, and potential predictive indicators. Longitudinal and multimodal evidence remains limited, restricting causal interpretation and the identification of clinically useful early markers. These limitations highlight the need for an integrated synthesis of clinical, retinal, neuroimaging, cognitive, molecular, and genetic evidence in T2DM.

Objective of the Review

This systematic review aimed to synthesize evidence on neurovascular, structural, metabolic, molecular, and genetic mechanisms associated with cognitive impairment in T2DM. The review included both studies directly assessing cognitive or cerebral outcomes and studies evaluating systemic microvascular or neurovascular abnormalities considered mechanistically relevant to diabetic neural injury. Indirect systemic markers were included to characterize the broader vascular milieu that may contribute to cerebral vulnerability, but they were interpreted separately from direct evidence of cognitive impairment.

## Review

Methodology

Study Design

This study was conducted as a systematic review and reported in accordance with the Preferred Reporting Items for Systematic Reviews and Meta-Analyses (PRISMA) 2020 framework. The review process comprised literature identification, duplicate removal, title and abstract screening, full-text eligibility assessment, data extraction, risk-of-bias assessment, and qualitative synthesis. Original human studies using observational, cross-sectional, longitudinal, neuroimaging, genetic, and Mendelian-randomization designs were considered when they evaluated neurovascular, structural, cognitive, metabolic, or molecular findings relevant to cognitive impairment in T2DM. Interventional studies and randomized controlled trials were not included because the review focused on observational and mechanistic evidence concerning cognitive and neurovascular alterations associated with T2DM. Owing to heterogeneity in study designs, populations, assessment methods, and outcome measures, the findings were synthesized narratively rather than quantitatively. The review protocol was not prospectively registered in PROSPERO or another systematic review registry.

Search Strategy

A structured literature search was conducted using PubMed/MEDLINE, Scopus, Web of Science, and Google Scholar to identify relevant studies examining cognitive impairment and associated neurovascular, structural, metabolic, molecular, and genetic alterations in T2DM. The search combined controlled vocabulary, where applicable, with free-text terms using the Boolean operators AND and OR. The core search strategy was as follows: ("type 2 diabetes mellitus" OR T2DM OR "type 2 diabetes") AND ("cognitive impairment" OR cognition OR "mild cognitive impairment" OR dementia) AND (neurovascular OR microvascular OR retinal OR neuroimaging OR "brain structure" OR "grey matter" OR "white matter" OR biomarker OR molecular OR genetic). The syntax was adapted to the search requirements of each database while retaining the same principal concepts. Reference lists of potentially eligible articles were also examined to identify additional relevant studies. The database searches identified 252 records, comprising 82 from PubMed/MEDLINE, 49 from Scopus, 45 from Google Scholar, and 76 from Web of Science. After removal of 41 duplicate records, 211 records proceeded to title and abstract screening, consistent with the PRISMA flow diagram.

Eligibility Criteria

Studies were selected based on predefined criteria, including population, diabetes status, study design, and relevant outcomes. Eligible participants were adults aged ≥18 years with T2DM, defined according to the diagnostic criteria reported in each primary study, including clinical diagnosis, medical records, or validated study classification. Mixed diabetes cohorts were included only when T2DM-specific data could be extracted separately, while studies restricted to type 1 diabetes were excluded. Prior stroke, dementia, major psychiatric illness, neurological disease, or severe sensory impairment were not imposed as independent review-level exclusion criteria; these conditions were considered according to the eligibility criteria of the individual primary studies. Eligible cognitive outcomes included global cognitive performance, mild cognitive impairment (MCI), dementia or cognitive-status classification, memory, attention, executive function, processing speed, verbal learning, and other objectively assessed cognitive domains using standardized or validated cognitive or neuropsychological instruments reported by the primary studies. Both MCI and dementia outcomes were eligible. Cerebral outcomes included gray and white matter volumes or atrophy, regional structural abnormalities, cerebral blood flow, regional homogeneity, and neurovascular coupling assessed by MRI or related neuroimaging techniques. Eligible retinal outcomes included retinal vessel or capillary density, retinal nerve fiber layer and ganglion cell complex measurements, retinal thickness, and electrophysiological evidence of retinal neurovascular or neurodegenerative dysfunction. Peripheral microcirculatory findings were eligible as indirect evidence of generalized diabetic microvascular injury rather than direct measures of cognitive impairment. Metabolic, molecular, and genetic outcomes included HbA1c and related metabolic factors, soluble low-density lipoprotein receptor-related protein 1 (sLRP1), LRP1-related biomarkers, and genetic variants or polygenic measures relevant to diabetic neurovascular or neurodegenerative mechanisms. A non-diabetic healthy control group was not mandatory because some studies compared cognitive subgroups within T2DM populations or used population-based designs; appropriate comparator groups were included where available. Observational, cross-sectional, longitudinal, neuroimaging, biomarker, genetic association, and Mendelian-randomization studies were eligible. Case reports, review articles, intervention studies, randomized controlled trials, non-English-language publications, and studies with outcomes unrelated to the predefined review scope were excluded. Only peer-reviewed studies meeting these predefined criteria were included in the final synthesis.

Study Selection

Following duplicate removal, titles and abstracts of retrieved records were screened independently by two reviewers according to the predefined eligibility criteria. Articles considered potentially relevant underwent independent full-text assessment by the same two reviewers. Eligibility assessment considered participant age and diabetes type, study design, cognitive outcomes, and relevant retinal, cerebral, vascular, structural, metabolic, molecular, or genetic outcomes. Studies were retained when they provided relevant evidence concerning neurovascular, structural, cognitive, metabolic, molecular, or genetic findings associated with cognitive impairment in T2DM. Disagreements regarding eligibility were resolved through discussion and consensus between the reviewers. Only studies satisfying all predefined eligibility criteria were included in the final narrative synthesis.

Data Extraction

Data were extracted independently by two reviewers using a standardized extraction form. The form was pilot-tested on a subset of eligible studies before formal extraction to ensure consistency and completeness. Extracted variables included study design, population characteristics, sample size, assessment methods, cognitive outcomes, neuroimaging and retinal findings, metabolic and molecular biomarkers, genetic measures, and principal findings. Any discrepancies between reviewers were resolved through discussion and consensus, with consultation of a third reviewer when necessary. Extracted data were subsequently organized into predefined thematic domains for qualitative synthesis.

Risk of Bias Assessment

The methodological quality and risk of bias of the included studies were assessed according to study design. The Newcastle-Ottawa Scale (NOS) was used for observational and neuroimaging studies [16], the Joanna Briggs Institute (JBI) Critical Appraisal Checklist was used for cross-sectional studies [17], and the ROBINS-I framework was applied to the Mendelian-randomization study [18]. RoB 2 was not used because no randomized controlled trials were included. Overall NOS judgments were derived from the pattern of ratings across the selection, comparability, and outcome domains, with greater methodological limitations resulting in a higher overall risk category. For JBI assessments, overall judgments were based on the pattern of fulfilled, unmet, and unclear checklist criteria, with particular consideration of participant selection, outcome measurement, and control of confounding; because the JBI checklist does not prescribe a universal numerical cutoff, these judgments were made qualitatively. For ROBINS-I, the overall risk-of-bias judgment was determined according to the highest level of concern identified across its assessed domains. Most included studies were judged to have moderate or moderate-to-high overall risk of bias, primarily because of cross-sectional designs, limited sample sizes, single-center recruitment, and incomplete adjustment for confounding variables.

Data Synthesis

A narrative synthesis was undertaken because meta-analysis was considered inappropriate due to substantial clinical and methodological heterogeneity across the included studies. The studies differed in design, participant characteristics, definitions of cognitive impairment, cognitive assessment instruments, neuroimaging and retinal techniques, biomarker measurements, genetic analyses, and reported outcome metrics. Several studies also examined distinct mechanistic domains rather than reporting comparable effect estimates for a common outcome, and some provided insufficient quantitative data for statistical pooling. These factors precluded meaningful quantitative synthesis without introducing substantial interpretive uncertainty. Findings were therefore organized into predefined thematic domains encompassing neurovascular and structural alterations and cognitive, metabolic, molecular, and genetic mechanisms. Similarities and differences among studies were examined descriptively to identify recurring patterns and associations relevant to cognitive impairment in T2DM.

Results

Search Results

The study selection process was conducted in accordance with PRISMA 2020. A total of 252 records were identified from four databases: PubMed/MEDLINE (n=82), Scopus (n=49), Google Scholar (n=45), and Web of Science (n=76). After removal of 41 duplicate records, 211 records remained for title and abstract screening. Of these, 164 records were excluded, leaving 47 full-text articles for eligibility assessment. Following full-text review, 37 articles were excluded, including 19 that did not meet the inclusion criteria, 13 with insufficient outcome data, and five non-English-language articles. Consequently, 10 studies fulfilled the predefined eligibility criteria and were included in the final qualitative synthesis. The complete study selection process is presented in the PRISMA 2020 flow diagram (Figure 1).

**Figure 1:**
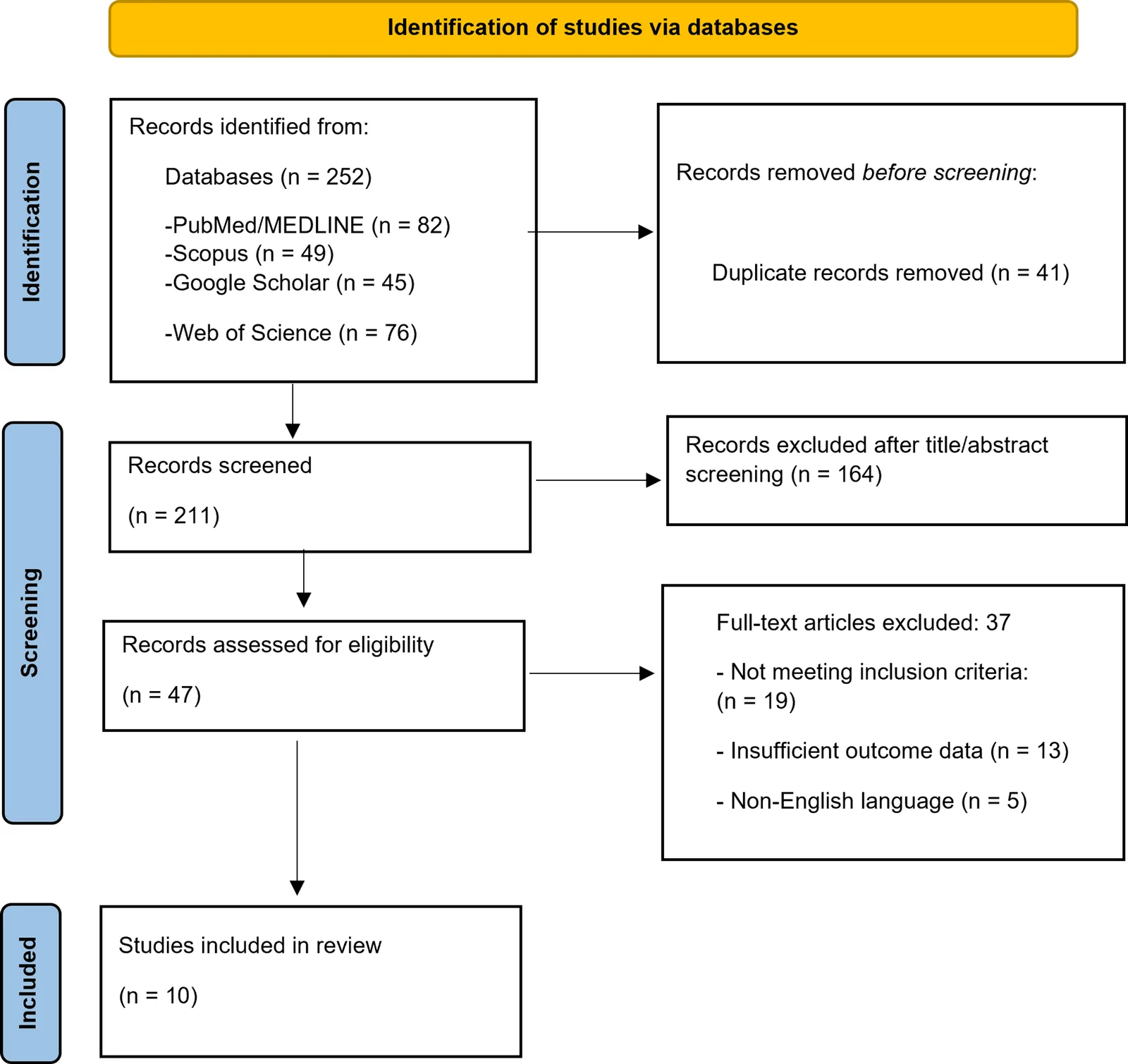
PRISMA Flow Diagram of the Study Selection Process

Study Characteristics

The studies incorporated were methodologically heterogeneous, and they, in combination, gave a multidimensional perspective of neurovascular and cognitive injury related to diabetes. They were cross-sectional, longitudinal, neuroimaging, biomarker, genetic association, and Mendelian-randomization designs. Most of the studies evaluated adults with type 2 diabetes, and some studies merely evaluated MCI or early microvascular dysfunction. The outcomes were retinal neurodegeneration, dermal microcirculatory, grey and white matter atrophy, neurovascular coupling, cognitive screening, and soluble LRP1, as demonstrated by the molecular findings. Neuropsychological measures, MRI, retinal imaging, laboratory assays, etc. were common measures that allowed the complementary structural, functional, and mechanistic domain assessment. Table 1 provides a summary of detailed characteristics.

**Table 1:** Main Characteristics of the Included Studies T2DM: type 2 diabetes mellitus; MCI: mild cognitive impairment; DR: diabetic retinopathy; MA: microalbuminuria; HbA1c: glycated hemoglobin; RNFL: retinal nerve fiber layer; SD-OCT: spectral-domain optical coherence tomography; ERG: electroretinography; OCTA: optical coherence tomography angiography; GCC: ganglion cell complex; MRI: magnetic resonance imaging; MMSE: Mini-Mental State Examination; MoCA: Montreal Cognitive Assessment; WAIS-III: Wechsler Adult Intelligence Scale, Third Edition; SFDI: spatial frequency domain imaging; LRP1: low-density lipoprotein receptor-related protein 1; sLRP1: soluble low-density lipoprotein receptor-related protein 1; DMN: default mode network; CBF: cerebral blood flow; ReHo: regional homogeneity; ASL: arterial spin labeling; HC: healthy control; T2DM-NC: type 2 diabetes mellitus with normal cognition; T2DM-MCI: type 2 diabetes mellitus with mild cognitive impairment; AUC: area under the curve

Study	Study design and population	Sample size	Assessment methods/key focus	Main findings
Zeng et al. [19]	Cross-sectional comparative study of patients with T2DM without clinically detectable diabetic retinopathy and healthy controls	128 participants: 66 T2DM without retinopathy and 62 healthy controls	Flicker electroretinography and optical coherence tomography angiography; retinal vessel density, ganglion cell complex, retinal nerve fibre layer, and radial peripapillary capillary density	Patients with T2DM showed reduced macular and peripapillary vessel density, thinner retinal nerve fibre layer, delayed electroretinographic implicit time, and reduced amplitude despite the absence of clinically detectable retinopathy.
Toprak et al. [20]	Cross-sectional study of patients with T2DM without diabetic retinopathy, stratified by microalbuminuria status, and matched non-diabetic controls	186 participants: 95 patients with T2DM, including 45 with microalbuminuria and 50 without microalbuminuria, and 91 controls	Spectral-domain optical coherence tomography; macular and peripapillary retinal thickness, diabetes duration, HbA1c, hypertension, and hyperlipidaemia	Inferior-temporal peripapillary retinal nerve fibre layer thickness was reduced in patients with diabetes. Poor glycaemic control and longer diabetes duration were associated with retinal thinning, whereas microalbuminuria was not significantly associated with retinal-layer measurements.
Li et al. [21]	Population-based cross-sectional study of Chinese adults aged ≥60 years; T2DM participants were assessed for MCI	3,246 participants screened; 256 participants with T2DM formed the principal diabetes sample, including 56 with MCI and 200 with normal cognition	MMSE, MoCA, digit span, associative learning, visual identification, verbal fluency, WAIS-III subtests, and auditory verbal learning	MCI prevalence among participants with T2DM was 21.8%. Depression was associated with increased MCI risk, education was protective, and T2DM-associated MCI involved impairment across several cognitive domains.
Murphy et al. [22]	One-year longitudinal observational imaging study of patients with diabetes and previous or current diabetic foot ulceration; two incident-ulcer cases were presented in detail	35 participants enrolled; 2 participants who developed ulcers during follow-up were included in the detailed case analysis	Monthly spatial frequency domain imaging of tissue oxygen saturation and superficial and deep dermal haemoglobin distribution	Neuropathic and neuroischaemic ulcers demonstrated distinct pre-ulcerative microcirculatory patterns. The findings were based on two detailed cases nested within the 35-participant cohort.
Li et al. [23]	Cross-sectional structural MRI study of early-onset MCI, T2DM, combined T2DM-MCI, and matched healthy controls	112 participants: 28 early-onset MCI, 28 T2DM, 28 T2DM-MCI, and 28 healthy controls	Three-dimensional high-resolution structural MRI; cortical and subcortical grey-matter volumes and default mode network structural covariance	Participants with T2DM-MCI showed greater grey-matter atrophy than participants with MCI or T2DM alone. Subcortical volume loss, particularly in the thalamus, putamen, and hippocampus, was associated with cognitive impairment.
Ware et al. [24]	Cross-sectional Mendelian-randomization analysis of adults aged ≥50 years from the 2010 Health and Retirement Study	10,322 participants	Self-reported T2DM, clinically validated cognitive classification, T2DM polygenic score, multivariable regression, and inverse-variance Mendelian randomization	T2DM was observationally associated with cognitive impairment, but Mendelian randomization did not demonstrate evidence of a causal association between T2DM and impaired cognition.
Li et al. [25]	Cross-sectional structural MRI study of patients with T2DM-MCI, T2DM with normal cognition, and matched healthy controls	90 participants: 30 T2DM-MCI, 30 T2DM with normal cognition, and 30 healthy controls	Neuropsychological testing, high-resolution structural MRI, automated regional white-matter volumetry, and receiver-operating-characteristic analysis	T2DM-MCI was associated with reduced white-matter volume in seven regions, mainly within the frontotemporal and limbic areas. White-matter volumes in the left posterior cingulate, precuneus, insula, and right rostral middle frontal gyrus showed potential value for detecting MCI.
Zhang et al. [26]	Five-year longitudinal MRI study of patients with T2DM and matched healthy controls	120 participants at baseline: 60 T2DM and 60 controls; 63 included in the final longitudinal analysis: 27 T2DM and 36 controls	Resting-state functional MRI, arterial spin labelling, regional homogeneity, cerebral blood flow, neurovascular-coupling ratio, and memory testing	Over five years, patients with T2DM demonstrated greater neurovascular-coupling deterioration in the left insula. Neurovascular-coupling abnormalities were associated with memory performance.
Suain Bon et al. [27]	Cross-sectional study of adults aged ≥60 years with T2DM attending specialist clinics in Hospital Kuala Lumpur	113 participants	Montreal Cognitive Assessment, Depression Anxiety Stress Scale, Mini-International Neuropsychiatric Interview, and logistic regression	Cognitive impairment prevalence was 46.9%. Ethnicity and education level were independently associated with cognitive impairment.
Cao et al. [28]	Cross-sectional genetic and biomarker study of Chinese patients with T2DM, divided according to cognitive status	166 participants: 60 with MCI and 106 with normal cognition	MoCA and neuropsychological testing, plasma soluble LRP1 measurement, HbA1c assessment, and LRP1 rs1799986 genotyping	Lower plasma soluble LRP1, higher HbA1c, insulin use, and hypertension were associated with MCI. The rs1799986 T allele was associated with better delayed verbal recall and may reduce susceptibility to MCI.

Risk of Bias Assessment

The possibility of bias was addressed using conventional appraisal methods suitable for observational findings, such as the NOS, JBI Critical Appraisal Checklist, and the ROBINS-I framework for non-randomized studies [16-18]. The vast majority of cases were of moderate quality of findings. Strengths included validated cognitive measures for the populations, objective imaging or biochemical measures, and well-defined populations of diabetics. The limitations were that the totality of the studies that were cross-sectional in nature, recruited in a single centre, were small, and they were unable to control for confounding variables and could not make inferences. The longitudinal and genetic studies reduced some of the bias concerns, yet they may still have selective sampling and confounding variables that will still be present in the ultimate body of findings. Table 2 demonstrates the risk of bias of the included studies.

**Table 2:** Risk of Bias Assessment of the Included Studies NOS: Newcastle-Ottawa Scale; JBI: Joanna Briggs Institute; ROBINS-I: Risk Of Bias In Non-randomized Studies of Interventions; MRI: Magnetic Resonance Imaging

Study	Study Design	Tool Used	Selection Bias	Performance Bias	Detection Bias	Confounding Bias	Overall Risk
Zeng et al. [19]	Cross-sectional comparative study	JBI Checklist	Low	Moderate	Low	Moderate	Moderate
Toprak et al. [20]	Cross-sectional study	JBI Checklist	Low	Moderate	Low	Moderate	Moderate
Li et al. [21]	Cross-sectional	JBI Checklist	Moderate	Moderate	Moderate	High	Moderate-High
Murphy et al. [22]	Longitudinal observational imaging study	NOS	Low	Moderate	Low	Moderate	Moderate
Li et al. [23]	Cross-sectional structural MRI study	JBI Checklist	Moderate	Moderate	Low	Moderate	Moderate
Ware et al. [24]	Cross-sectional Mendelian-randomisation analysis	ROBINS-I	Low	Low	Low	Low	Low
Li et al. [25]	Cross-sectional structural MRI study	JBI Checklist	Moderate	Moderate	Low	Moderate	Moderate
Zhang et al. [26]	Longitudinal MRI	NOS	Low	Low	Low	Moderate	Low-Moderate
Suain Bon et al. [27]	Cross-sectional	JBI Checklist	Moderate	Moderate	Moderate	High	Moderate-High
Cao et al. [28]	Cross-sectional genetic and biomarker study	JBI Checklist	Moderate	Moderate	Moderate	High	Moderate-High

Neurovascular and Structural Pathways

The concluding study demonstrates that the correlation between neurovascular and structural processes is complicated with respect to the impact of T2DM on cognition. These initial malformations of the retina, neurovascular changes, and neurodegeneration of the retina imply that diabetes may cause neural and vascular tissue damage before the onset of retinopathy. According to other brain-based findings, the memory, executive, and attention areas degenerate the grey mass, damage the white mass, and change the neurovascular linkage. This predisposition to systemic dysfunction of the microcirculation is further lengthened by the defects of the dermal microcirculation. These data, together with the published literature, suggest a common pathophysiologic pathway of chronic hyperglycemia, maladaptation of dysperfusion, and tissue-level neurovascular maladaptation that predisposes neural vulnerability and cognitive impairment. The frequency of neurovascular and structural findings reported across the included studies is presented in Figure 2.

**Figure 2:**
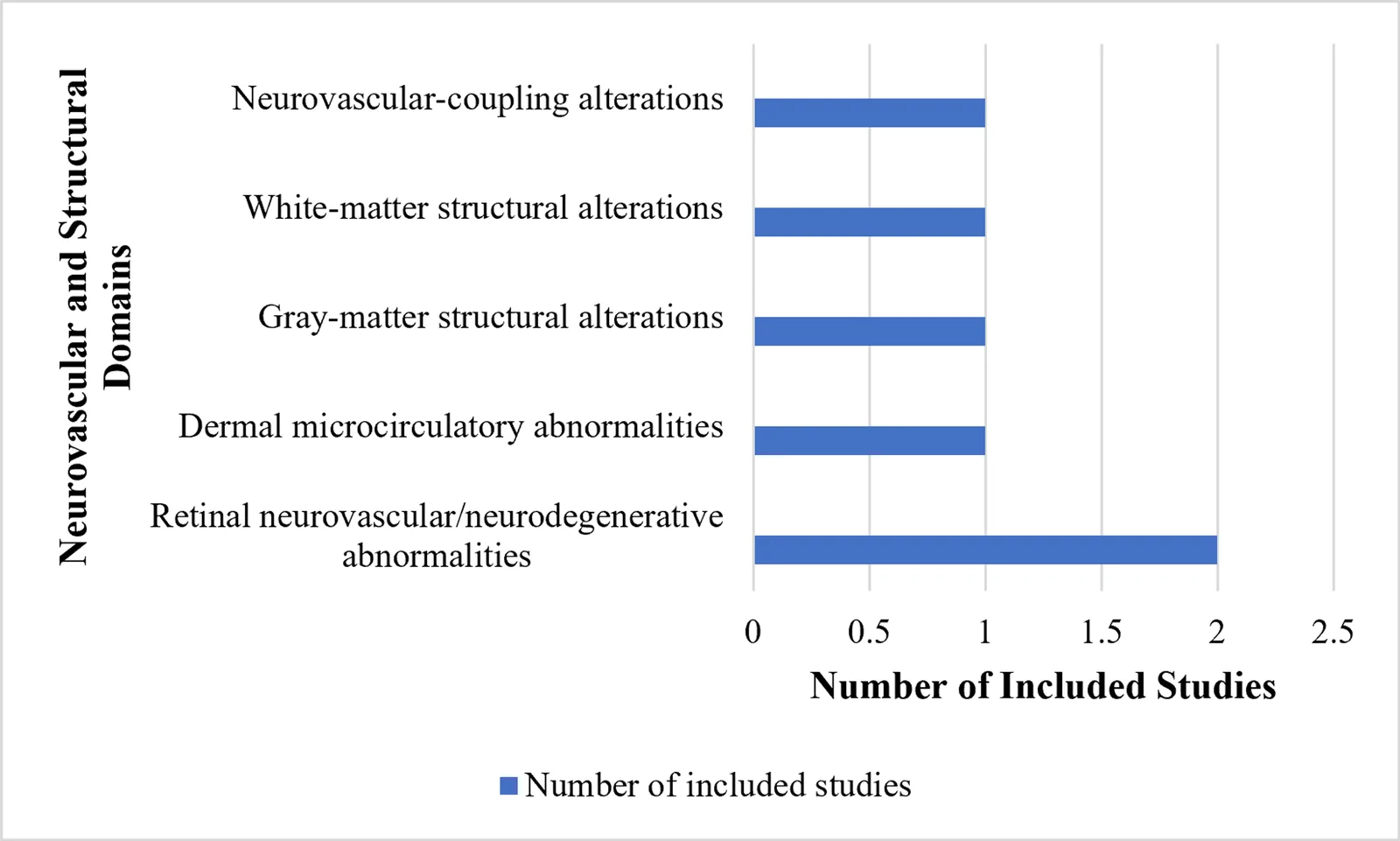
Frequency of Neurovascular and Structural Findings Across the Included Studies Source: Refs. [19,20,22,23,25,26]

Cognitive and Molecular Mechanisms

The studies that have been included also indicate that cognitive decline caused by diabetes is dependent on metabolic and molecular processes, in addition to structural damage. Higher HbA1c, as a specific marker, was found to be a factor in suboptimal glycemic control over and over again, which led to cognitive impairment. There are other biomarkers and genetic data that indicate a mechanistic role in pathways of soluble LRP1, amyloid processing, and susceptibility polymorphisms. The epidemiologic data showed that older diabetic populations were characterised by high rates of cognitive impairment, and that diabetes as a causal factor was not strongly Mendelianly randomized, and intermediate processes may be more important. All these, combined, indicate a multifactorial model of metabolism, vascular injury, neurodegeneration, and cognition. The frequency of cognitive, metabolic, molecular, and genetic findings reported across the included studies is presented in Figure 3.

**Figure 3:**
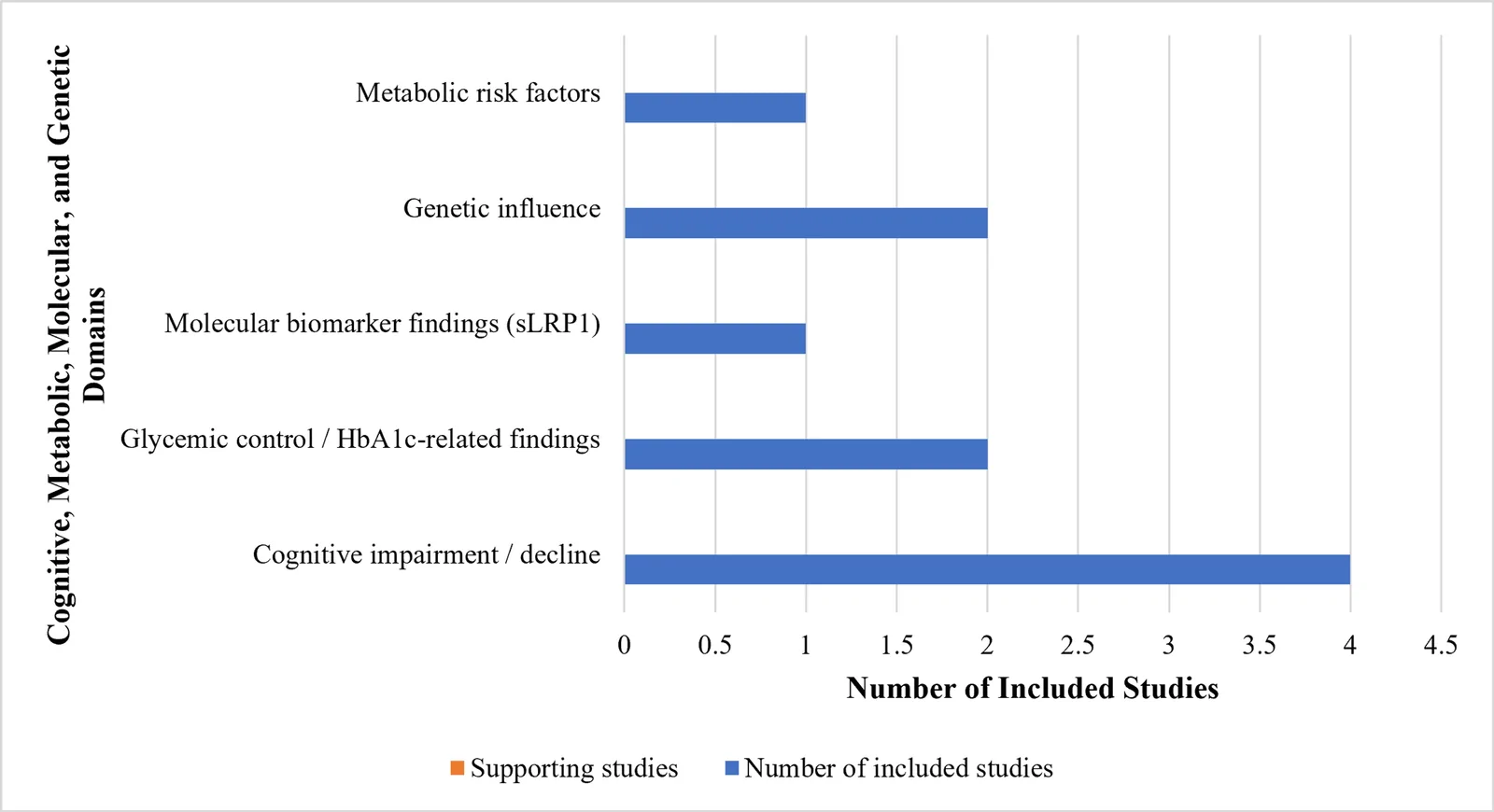
Frequency of Cognitive, Metabolic, Molecular, and Genetic Findings Across the Included Studies Source: Refs. [20,21,24,27,28]

Discussion

Principal Findings

This systematic review synthesizes evidence on the neurovascular, structural, metabolic, molecular, and genetic factors associated with cognitive impairment in T2DM. The findings suggest that cognitive vulnerability in T2DM is associated with abnormalities across both peripheral and central neurovascular systems. Retinal neurovascular dysfunction and neurodegenerative changes have been reported in individuals with diabetes even before clinically detectable diabetic retinopathy, supporting the possibility that retinal abnormalities may reflect early neurovascular involvement [27,28]. Neuroimaging studies have also demonstrated gray- and white-matter structural alterations, particularly in regions involved in memory, attention, and executive function [29,30]. Longitudinal evidence further indicates deterioration in neurovascular coupling, supporting an association between impaired cerebral perfusion regulation and cognitive function [31].

Metabolic and molecular abnormalities provide additional mechanisms through which T2DM may be associated with cognitive impairment. Higher glycated hemoglobin (HbA1c), reflecting poorer glycemic control, has been associated with cognitive dysfunction and structural brain alterations [32]. Chronic hyperglycemia may contribute to oxidative stress, inflammation, advanced glycation end-product accumulation, and impaired neuronal function. Altered insulin signaling may also affect neuronal metabolism and synaptic function [33]. Molecular findings involving soluble low-density lipoprotein receptor-related protein 1 (sLRP1) suggest a potential relationship between metabolic dysfunction, amyloid processing, and cognitive vulnerability [34]. Genetic findings indicate that specific polymorphisms may influence susceptibility to cognitive impairment, although the available evidence remains limited. Mendelian-randomization evidence did not establish a direct causal relationship between T2DM and impaired cognition, indicating that intermediary vascular, metabolic, and neurodegenerative pathways may contribute to the observed association.

Clinical Implications

The findings highlight the potential importance of recognizing cognitive dysfunction as part of the broader systemic consequences of T2DM. Retinal abnormalities, structural neuroimaging findings, neurovascular-coupling changes, metabolic indicators, and molecular biomarkers may provide complementary information for identifying individuals at increased cognitive risk. These findings do not yet support routine use of any single retinal, imaging, or molecular marker as a diagnostic test; however, they indicate areas that warrant further clinical validation. Management of modifiable vascular and metabolic risk factors remains clinically relevant. Optimizing glycemic control and addressing hypertension and other metabolic abnormalities may support overall vascular and neurological health [35]. Cognitive assessment may also be considered in individuals with T2DM who have clinical concerns regarding memory, attention, or executive function, particularly when additional vascular or metabolic risk factors are present.

Strengths

A strength of this review is the integration of evidence from multiple complementary domains, including retinal assessment, cerebral neuroimaging, cognitive testing, metabolic parameters, molecular biomarkers, and genetic analyses. The inclusion of cross-sectional, longitudinal, neuroimaging, biomarker, genetic, and Mendelian-randomization evidence allowed the relationship between T2DM and cognitive impairment to be examined from several mechanistic perspectives. The review also used predefined eligibility criteria, independent study selection and data extraction by two reviewers, study-design-appropriate risk-of-bias assessment, and PRISMA-based reporting. Narrative synthesis was used to account for substantial methodological and outcome heterogeneity rather than combining clinically dissimilar findings quantitatively.

Limitations and Future Directions

The findings of this review should be interpreted in light of several limitations. Most of the included studies were cross-sectional, which limits assessment of temporal relationships and prevents firm conclusions regarding causality between T2DM and cognitive impairment. Heterogeneity in study designs, populations, assessment methods, and outcome measures may also have affected the comparability and consistency of findings across studies. Several investigations included relatively small or population-specific samples, which may limit the generalizability of their results. Residual confounding may also have influenced some reported associations because lifestyle factors, comorbid conditions, medication use, and other relevant variables were not uniformly adjusted across studies. Differences in neuroimaging techniques and cognitive assessment instruments further contributed to variability in the available evidence. A quantitative meta-analysis was not performed because the included studies differed substantially in design, populations, assessment methods, and reported outcomes, which precluded meaningful statistical pooling. Publication bias could therefore not be formally assessed using methods such as funnel-plot analysis or statistical tests, and the possibility of selective publication of positive findings cannot be excluded. The review protocol was not prospectively registered in PROSPERO or another systematic review registry, which is also acknowledged as a methodological limitation.

Future research should prioritize well-designed longitudinal and interventional studies to clarify temporal relationships and disease progression. Larger, multicentre studies involving more diverse populations are needed to improve generalizability and reduce the influence of population-specific findings. Multimodal approaches integrating neuroimaging, retinal assessment, cognitive testing, molecular biomarkers, and genetic profiling may provide a more comprehensive understanding of the mechanisms linking T2DM with cognitive impairment. Greater standardization of assessment methods and outcome measures would improve comparability across studies and may facilitate future quantitative synthesis. Further research is also needed to validate potential early biomarkers and to determine whether targeted management of modifiable metabolic and vascular risk factors can reduce the risk or progression of cognitive impairment in individuals with T2DM.

## Conclusions

Cognitive impairment in T2DM is associated with interconnected neurovascular, structural, metabolic, and molecular alterations. Retinal and cerebral vascular changes, gray and white matter abnormalities, impaired glycemic regulation, and molecular disturbances may contribute to cognitive vulnerability. Early recognition of these changes, together with management of modifiable vascular and metabolic risk factors, may support improved risk stratification and long-term neurological health. Further longitudinal and multimodal studies are needed to clarify causal pathways and validate clinically useful biomarkers.
